# Supplementary material for: National implementation trial of BeUpstanding™: an online initiative for workers to sit less and move more
Source: Int J Behav Nutr Phys Act. 2024 Sep 30;21:111. doi: 10.1186/s12966-024-01652-0 (PMC11441226; doi:10.1186/s12966-024-01652-0)
Supplement: Supplementary file 1 — Supplementary Material 1 [file 12966_2024_1652_MOESM1_ESM.docx]

**Additional Files**

**Additional File 1:** TREND checklist

| **Paper Section/Topic** | **Item No.** | | **Descriptor** | Reported? | |
| --- | --- | --- | --- | --- | --- |
|  |  |  |  |  | Pg # |
| **TITLE and ABSTRACT** | | | |  |  |
| Title and Abstract | 1 | | - Information on how units were allocated to interventions | NA |  |
|  |  | | - Structured abstract recommended | ✓ | 3 |
|  |  | | - Information on target population or study sample | ✓ | 3 |
| **INTRODUCTION** | | | |  |  |
| Background | 2 | - Scientific background and explanation of rationale | | ✓ | 5 |
|  |  | - Theories used in designing behavioral interventions | | ✓ | 5 |
| **METHODS** | | | |  |  |
| Participants | 3 | | - Eligibility criteria for participants, including criteria at different levels in recruitment/sampling plan (e.g., cities, clinics, subjects) | ✓ | 6, 7 |
|  |  | | - Method of recruitment (e.g., referral, self-selection), including the sampling method if a systematic sampling plan was implemented | ✓ | 6, 7 |
|  |  | | - Recruitment setting | ✓ | 7 |
|  |  | | - Settings and locations where the data were collected | ✓ | 9 |
| Interventions | 4 | | - Details of the interventions intended for each study condition and how and when they were actually administered, specifically including: | ✓ | All 8 |
|  |  | | - - Content: what was given? | ✓ |  |
|  |  | | - - Delivery method: how was the content given? | ✓ |  |
|  |  | | - - Unit of delivery: how were subjects grouped during delivery? | ✓ |  |
|  |  | | - - Deliverer: who delivered the intervention? | ✓ |  |
|  |  | | - - Setting: where was the intervention delivered? | ✓ |  |
|  |  | | - - Exposure quantity and duration: how many sessions or episodes or events were intended to be delivered? How long were they intended to last? | ✓ |  |
|  |  | | - - Time span: how long was it intended to take to deliver the intervention to each unit? | ✓ |  |
|  |  | | - - Activities to increase compliance or adherence (e.g., incentives) | ✓ |  |
| Objectives | 5 | | - Specific objectives and hypotheses | ✓ | 5 |
| Outcomes | 6 | | - Clearly defined primary and secondary outcome measures | ✓ | 9-11 |
|  |  | | - Methods used to collect data and any methods used to enhance the quality of measurements | ✓ | 9-11 |
|  |  | | - Information on validated instruments such as psychometric and biometric properties | X in protocol |  |
| Sample size | 7 | | - How sample size was determined and, when applicable, explanation of any interim analyses and stopping rules | ✓ | 11 |
| Assignment method | 8 | | - Unit of assignment (the unit being assigned to study condition, e.g., individual, group, community) | NA |  |
|  |  | | - Method used to assign units to study conditions, including details of any restriction (e.g., blocking, stratification, minimization) | NA |  |
|  |  | | - Inclusion of aspects employed to help minimize potential bias induced due to non-randomization (e.g., matching) | ✓ | 12 |
| Blinding (masking) | 9 | | - Whether or not participants, those administering the interventions, and those assessing the outcomes were blinded to study condition assignment; if so, statement regarding how the blinding was accomplished and how it was assessed | ✓ | 9 |
| Unit of Analysis | 10 | | - Description of the smallest unit that is being analysed to assess intervention effects (e.g., individual, group, or community) | ✓ | 11 |
|  |  | | - If the unit of analysis differs from the unit of assignment, the analytical method used to account for this (e.g., adjusting the standard error estimates by the design effect or using multilevel analysis) | ✓ | 11-12 |
| Statistical methods | 11 | | - Statistical methods used to compare study groups for primary methods outcome(s), including complex methods for correlated data | ✓ | 11-12 |
|  |  |  | - Statistical methods used for additional analyses, such as subgroup analyses and adjusted analysis | ✓ | 11-12 |
|  |  |  | - Methods for imputing missing data, if used | ✓ | 11-12 |
|  |  |  | - Statistical software or programs used | ✓ | 12 |
| **RESULTS** | | | |  |  |
| Participant flow | 12 | | - Flow of participants through each stage of the study: enrollment, assignment, allocation and intervention exposure, follow-up, analysis (a diagram is strongly recommended) | ✓ | Fig.1 for all |
|  |  | | - - Enrollment: the numbers of participants screened for eligibility, found to be eligible or not eligible, declined to be enrolled, and enrolled in the study | ✓ |  |
|  |  | | - - Assignment: the numbers of participants assigned to a study condition | ✓ |  |
|  |  | | - - Allocation and intervention exposure: the number of participants assigned to each study condition and the number of participants who received each intervention | ✓ |  |
|  |  | | - - Follow-up: the number of participants who completed the follow-up or did not complete the follow-up (i.e., lost to follow-up), by study condition | ✓ |  |
|  |  | | - - Analysis: the number of participants included in or excluded from the main analysis, by study condition | ✓ |  |
|  |  | | - Description of protocol deviations from study as planned, along with reasons | ✓ | 8 |
| Recruitment | 13 | | - Dates defining the periods of recruitment and follow-up | ✓ |  |
| Baseline data | 14 | | - Baseline demographic and clinical characteristics of participants in each study condition | ✓ | Tables 1, 2 |
|  |  | | - Baseline characteristics for each study condition relevant to specific disease prevention research | NA |  |
|  |  | | - Baseline comparisons of those lost to follow-up and those retained, overall and by study condition | ✓ | AF 4-7 |
|  |  | | - Comparison between study population at baseline and target population of interest | NA |  |
| Baseline equivalence | 15 | | - Data on study group equivalence at baseline and statistical methods used to control for baseline differences | NA |  |
| Numbers analyzed | 16 | | - Number of participants (denominator) included in each analysis for each study condition, particularly when the denominators change for different outcomes; statement of the results in absolute numbers when feasible | ✓ | Tables 1-5 |
|  |  | | - Indication of whether the analysis strategy was “intention to treat” or, if not, description of how non-compliers were treated in the analyses | ✓ | 12 |
| Outcomes and estimation | 17 | | - For each primary and secondary outcome, a summary of results for each estimation study condition, and the estimated effect size and a confidence interval to indicate the precision | ✓ | Fig 2, Table 5 |
|  |  | | - Inclusion of null and negative findings | ✓ | 12-18 |
|  |  | | - Inclusion of results from testing pre-specified causal pathways through which the intervention was intended to operate, if any | ✓ | 15-16 |
| Ancillary analyses | 18 | | - Summary of other analyses performed, including subgroup or restricted analyses, indicating which are pre-specified or exploratory | ✓ | 15-17 |
| Adverse events | 19 | | - Summary of all important adverse events or unintended effects in each study condition (including summary measures, effect size estimates, and confidence intervals) | ✓ | 17-18 |
| **DISCUSSION** | | | |  |  |
| Interpretation | 20 | | - Interpretation of the results, taking into account study hypotheses, sources of potential bias, imprecision of measures, multiplicative analyses, and other limitations or weaknesses of the study | ✓ | 19-23 |
|  |  | | - Discussion of results taking into account the mechanism by which the intervention was intended to work (causal pathways) or alternative mechanisms or explanations | ✓ | 21 |
|  |  | | - Discussion of the success of and barriers to implementing the intervention, fidelity of implementation | ✓ | 20 |
|  |  | | - Discussion of research, programmatic, or policy implications | ✓ | 19-24 |
| Generalizability | 21 | | - Generalizability (external validity) of the trial findings, taking into account the study population, the characteristics of the intervention, length of follow-up, incentives, compliance rates, specific sites/settings involved in the study, and other contextual issues | ✓ | 20 |
| Overall evidence | 22 | | - General interpretation of the results in the context of current evidence and current theory | ✓ | 19-24 |

*From:*  Des Jarlais, D. C., Lyles, C., Crepaz, N., & the Trend Group (2004). Improving the reporting quality of nonrandomized evaluations of behavioral and public health interventions: The TREND statement. *American Journal of Public Health*, 94, 361-366. For more information, visit: <http://www.cdc.gov/trendstatement/>

**Additional File 2:** The TIDieR (Template for Intervention Description and Replication) Checklist

| **Item no.** | **Item** | **Where located **** | |
| --- | --- | --- | --- |
|  |  | Primary paper  (page or appendix  number) | Other ^†^ (details) |
|  | **BRIEF NAME** |  |  |
| **1.** | Provide the name or a phrase that describes the intervention. | 3 |  |
|  | **WHY** |  |  |
| **2.** | Describe any rationale, theory, or goal of the elements essential to the intervention. | 5 | Protocol paper |
|  | **WHAT** |  |  |
| **3.** | Materials: Describe any physical or informational materials used in the intervention, including those provided to participants or used in intervention delivery or in training of intervention providers. Provide information on where the materials can be accessed (e.g. online appendix, URL). | 8 | www.beupstanding.com.au |
| **4.** | Procedures: Describe each of the procedures, activities, and/or processes used in the intervention, including any enabling or support activities. | 8 | Protocol paper |
|  | **WHO PROVIDED** |  |  |
| **5.** | For each category of intervention provider (e.g. psychologist, nursing assistant), describe their expertise, background and any specific training given. | 9 |  |
|  | **HOW** |  |  |
| **6.** | Describe the modes of delivery (e.g. face-to-face or by some other mechanism, such as internet or telephone) of the intervention and whether it was provided individually or in a group. | 8 |  |
|  | **WHERE** |  |  |
| **7.** | Describe the type(s) of location(s) where the intervention occurred, including any necessary infrastructure or relevant features. | 8 | Protocol paper |
|  | **WHEN and HOW MUCH** |  |  |
| **8.** | Describe the number of times the intervention was delivered and over what period of time including the number of sessions, their schedule, and their duration, intensity or dose. | 8 | Protocol paper |
|  | **TAILORING** |  |  |
| **9.** | If the intervention was planned to be personalised, titrated or adapted, then describe what, why, when, and how. | 8 | Protocol paper |
|  | **MODIFICATIONS** |  |  |
| **10.^ǂ^** | If the intervention was modified during the course of the study, describe the changes (what, why, when, and how). | 8 |  |
|  | **HOW WELL** |  |  |
| **11.** | Planned: If intervention adherence or fidelity was assessed, describe how and by whom, and if any strategies were used to maintain or improve fidelity, describe them. | 8, 10, 15, Table 3 |  |
| **12.^ǂ^** | Actual: If intervention adherence or fidelity was assessed, describe the extent to which the intervention was delivered as planned. | 10, 15, Table 3 |  |

**Protocol paper:** Healy GN, Goode AD, Abbott A, et al., Supporting Workers to Sit Less and Move More Through the Web-Based BeUpstanding Program: Protocol for a Single-Arm, Repeated Measures Implementation Study. JMIR Res Protoc. 2020 May 4;9(5):e15756. doi: 10.2196/15756

**Additional File 3:** Example of resources provided for work from home

**
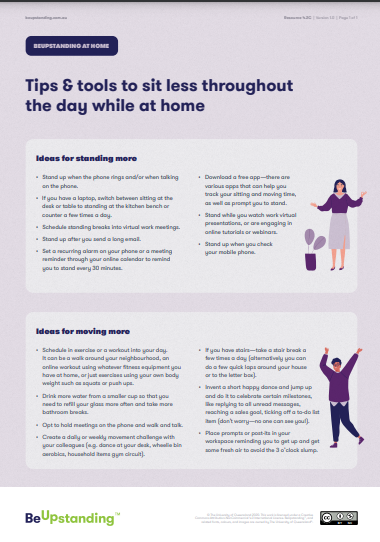
**

**Additional File 4:** What those who did and did not participate in the trial hoped the program would achieve and the association of these aims with the odds of participation ^a^

|  | In Trial (n=116) | Not in trial (n=729) | Association | |
| --- | --- | --- | --- | --- |
|  | n(%) | n(%) | OR (95%CI) | p |
| Improve physical health and wellbeing | 13 (11.2%) | 80 (11.0%) | 1.02 (0.55, 1.91) | 0.941 |
| Improve sitting / physical behaviours | 44 (37.9%) | 234 (32.1%) | 1.29 (0.86, 1.94) | 0.215 |
| Improve culture | 17 (14.7%) | 59 (8.1%) | 1.95 (1.09, 3.48) | **0.024** |
| Improve connectedness | 16 (13.8%) | 58 (8.0%) | 1.85 (1.02, 3.35) | **0.041** |
| Improve productivity | 15 (12.9%) | 37 (5.1%) | 2.78 (1.47, 5.24) | **0.002** |
| Improve workplace health and safety alignment | 12 (10.3%) | 67 (9.2%) | 1.14 (0.60, 2.18) | 0.692 |
| Improve awareness of sitting / physical behaviours | 11 (9.5%) | 80 (11.0%) | 0.85 (0.44, 1.65) | 0.631 |
| Improve happiness / morale | 11 (9.5%) | 66 (9.1%) | 1.05 (0.54, 2.06) | 0.881 |
| Improve awareness of ergonomics | 1 (0.9%) | 40 (5.5%) | 0.15 (0.02, 1.10) | 0.062 |
| Improve psychological health and wellbeing | 59 (50.9%) | 302 (41.4%) | 1.46 (0.99, 2.17) | 0.057 |
| Test the program for possible wider use | 4 (3.4%) | 16 (2.2%) | 1.59 (0.52, 4.85) | 0.413 |
| Improve awareness of health | 3 (2.6%) | 59 (8.1%) | 0.30 (0.09, 0.98) | **0.046** |
| Improve awareness of work practices | 2 (1.7%) | 6 (0.8%) | 2.11 (0.42, 10.60) | 0.363 |
| Improve conditions for those working from home | 2 (1.7%) | 28 (3.8%) | 0.44 (0.10, 1.87) | 0.265 |
| Enact organisational values / excellence | 2 (1.7%) | 6 (0.8%) | 2.11 (0.42, 10.60) | 0.363 |
| Improve awareness in general | 4 (3.4%) | 12 (1.6%) | 2.13 (0.68, 6.73) | 0.196 |
| Complement existing programs | 1 (0.9%) | 23 (3.2%) | 0.27 (0.04, 2.00) | 0.198 |
| Aim not related to the program | 1 (0.9%) | 15 (2.1%) | 0.41 (0.05, 3.16) | 0.395 |
| Address the impact of COVID-19 | 0 (0.0%) | 6 (0.8%) | - | - |
| Provide an alternative to existing programs | 0 (0.0%) | 7 (1.0%) | - | - |
| Miscellaneous other aims | 0 (0.0%) | 15 (2.1%) | - | - |
| No aim or irrelevant response | 0 (0.0%) | 26 (3.6%) | - | - |

^a^ Logistic regression models

**Additional File 5:** Odds of workplaces participating in the trial by champion, team, and workplace / organisational characteristics

| Variable | Comparison | OR (95%CI) | p ^a^ |
| --- | --- | --- | --- |
| ***Champion characteristics*** |  |  |  |
| Location (State) - collapsed | VIC vs NSW&ACT | 1.04 (1.94, 0.00) | <0.001 |
|  | QLD vs NSW&ACT | 2.30 (1.38, 3.85) |  |
|  | SA vs NSW&ACT | 1.00 (0.43, 2.33) |  |
|  | WA/NT/TAS vs NSW&ACT | 0.69 (0.31, 1.53) |  |
| Postcode SES - State Percentile (1-100) ^b^ | *per 10 percentiles* | 0.99 (0.92, 1.07) | 0.827 |
| Postcode SES - Australian Percentile (1-100) ^b^ | *per 10 percentiles* | 0.99 (0.92, 1.06) | 0.727 |
| Age | *per 10 years* | 0.99 (0.82, 1.18) | 0.886 |
| Female | Yes vs No | 1.21 (0.76, 1.90) | 0.422 |
| Job Classification | Middle management vs employee | 0.88 (0.57, 1.35) | 0.828 |
|  | Upper management vs employee | 0.90 (0.50, 1.59) |  |
| Has OHS role | Yes vs No | 0.60 (0.39, 0.91) | 0.017 |
| Has prior WPHP Training | Yes vs No | 0.76 (0.51, 1.13) | 0.172 |
| Has prior WPHP Experience | Yes vs No | 0.72 (0.48, 1.08) | 0.115 |
| Referred by … (multiples apply) |  |  |  |
| Article / Publication / Newsletter | Yes vs No | 0.56 (0.32, 0.98) | 0.041 |
| Internet / Website | Yes vs No | 0.77 (0.49, 1.21) | 0.249 |
| Seminar / Presentation / Conference | Yes vs No | 1.97 (1.07, 3.64) | 0.029 |
| Social Media / TV / Radio | Yes vs No | 0.80 (0.28, 2.32) | 0.687 |
| Colleague | Yes vs No | 2.29 (1.48, 3.52) | <0.001 |
| Other word of mouth | Yes vs No | 1.31 (0.70, 2.47) | 0.400 |
| General Workplace | Yes vs No | 0.63 (0.08, 4.93) | 0.656 |
| Health & Safety Organisation | Yes vs No | 0.59 (0.14, 2.56) | 0.482 |
| Email | Yes vs No | 0.18 (0.02, 1.31) | 0.090 |
| Other source | Yes vs No | 0.92 (0.35, 2.40) | 0.866 |
| ***Team characteristics*** |  |  |  |
| Team Size category | 11-20 vs 1-10 | 1.90 (1.09, 3.32) | 0.079 |
|  | >20 vs 1-10 | 1.31 (0.79, 2.18) |  |
| Regional / remote staff included | Yes vs No | 1.08 (0.72, 1.63) | 0.713 |
| Call centre staff included | Yes vs No | 0.38 (0.16, 0.89) | 0.026 |
| Currently experiencing WPHP Program | Yes vs No | 0.82 (0.53, 1.28) | 0.384 |
| Interest in health (1-5) | *per 1 point* | 1.10 (0.86, 1.41) | 0.434 |
| Motivation to sit less (1-5) | *per 1 point* | 1.16 (0.89, 1.50) | 0.267 |
| Stress (1-5) | *per 1 point* | 1.17 (0.85, 1.60) | 0.341 |
| ***Workplace / organisational characteristics*** |  |  |  |
| Small-medium enterprise | Yes vs No | 0.50 (0.32, 0.79) | 0.003 |
| Blue collar workplace | Yes vs No | 0.74 (0.49, 1.12) | 0.157 |
| Sector | Non-profit vs public | 0.69 (0.39, 1.21) | 0.001 |
|  | Private vs public | 0.45 (0.29, 0.69) |  |
| COVID-19 impact level | General vs none | 2.45 (1.03, 5.79) | <0.001 |
|  | High vs none | 5.30 (2.18, 12.85) |  |
| Organisational readiness - context (1-5) | *per 1 point* | 1.42 (0.94, 2.16) | 0.098 |
| Organisational readiness - change effort (0-1) | *per 1 point* | 1.50 (0.77, 2.93) | 0.233 |
| Organisational readiness - change efficacy (1-5) | *per 1 point* | 1.52 (1.09, 2.12) | 0.014 |
| Industry (versus Administrative & Support Services | Agriculture / Forestry and Fishing | 1.38 (0.33, 5.77) | 0.031 |
|  | Construction + Mining & Quarries | 0.60 (0.18, 2.05) |  |
|  | Education & Training | 0.33 (0.10, 1.12) |  |
|  | Electricity / Gas / Water and Waste Services | 0.88 (0.27, 2.81) |  |
|  | Financial and Insurance Services | 0.90 (0.22, 3.62) |  |
|  | Health Care and Social Assistance | 0.98 (0.37, 2.59) |  |
|  | Manufacturing | 0.70 (0.23, 2.16) |  |
|  | Other Services | 0.59 (0.19, 1.87) |  |
|  | Professional / Scientific and Technical Services | 1.52 (0.56, 4.17) |  |
|  | Public Administration and Safety | 0.32 (0.10, 1.03) |  |
|  | Retail + Wholesale Trade | 0.33 (0.06, 1.80) |  |
|  | All other industries ^c^ | 0.53 (0.16, 1.74) |  |

^a^ p-value for collective test of all parameters

^b^ SES = Socioeconomic Status (Index of Relative Socioeconomic Advantage and Disadvantage

^c^ The other remaining industries included were: Arts and Recreation Services; Accommodation and Food Services; Information Media and Telecommuncations; and, Rental Hiring and Real Estate Services.

**Additional File 6:** Odds of team participating in post-program evaluation ^a^

| **Characteristic** | **Category** | **OR (95%CI)** | **p** |
| --- | --- | --- | --- |
| ***Champion characteristics*** |  |  |  |
| Location (State) (vs New South Wales & Australian Capital Territory) | Victoria | 4.37 (0.45, 42.08) | 0.343 |
|  | Queensland | 0.72 (0.22, 2.39) |  |
|  | South Australia | 0.42 (0.07, 2.53) |  |
|  | All other States & Territories | 2.19 (0.21, 22.34) |  |
| Postcode SES ^b^ |  |  |  |
| State Percentile | *per 10 percentiles* | 1.12 (0.95, 1.32) | 0.191 |
| Australian Percentile | *per 10 percentiles* | 1.12 (0.95, 1.34) | 0.181 |
| Age | *per 10 years* | 1.40 (0.91, 2.16) | 0.125 |
| Female | yes vs no | 0.48 (0.13, 1.80) | 0.275 |
| Job Classification (vs employee) | Middle management | 0.78 (0.29, 2.09) | 0.252 |
|  | Upper management | 4.81 (0.57, 40.96) |  |
| Occupational Health & Safety role | yes vs no | 1.80 (0.69, 4.66) | 0.230 |
| Prior workplace health promotion training | yes vs no | 1.68 (0.65, 4.32) | 0.285 |
| Prior workplace health promotion experience | yes vs no | 1.64 (0.57, 4.67) | 0.357 |
| ***Team characteristics*** |  |  |  |
| Team Size category | 11-20 vs 1-10 | 1.10 (0.34, 3.56) | 0.580 |
|  | >20 vs 1-10 | 1.78 (0.57, 5.53) |  |
| Regional/remote staff included | yes vs no | 0.36 (0.14, 0.95) | **0.038** |
| Call-centre staff included | yes vs no | 0.14 (0.02, 0.81) | **0.028** |
| Interest in health (1-5) | *per 1 point* | 1.45 (0.74, 2.85) | 0.284 |
| Motivation to sit less (1-5) | *per 1 point* | 0.87 (0.45, 1.66) | 0.668 |
| Stress (1-5) | *per 1 point* | 0.70 (0.30, 1.66) | 0.417 |
| Currently receiving workplace health promotion program | yes vs no | 1.32 (0.43, 4.04) | 0.632 |
| ***Workplace / organisational characteristics*** |  |  |  |
| Small-medium enterprise | yes vs no | 10.54 (1.34, 83.10) | 0.025 |
| Sector | Non-profit vs public | 6.50 (0.78, 54.19) | 0.115 |
|  | Private vs public | 2.17 (0.74, 6.33) |  |
| Blue-collar workplace | yes vs no | 0.62 (0.24, 1.63) | 0.335 |
| Main Industry of Organisation | Manufacturing | 0.46 (0.09, 2.50) | 0.283 |
| (vs Healthcare and Social Assistance) | Professional | 1.20 (0.29, 4.94) |  |
|  | Administrative Services + Public Administration Education + Other + Financial + Arts + Accommodation | 2.00 (0.54, 7.43) |  |
|  | Agriculture, Construction, Retail + Wholesale + Electricity | 3.46 (0.59, 20.21) |  |
| Highly impacted by COVID-19 | yes vs no | 0.27 (0.10, 0.72) | 0.009 |
| Organisational readiness |  |  |  |
| Context score | *per 1 point* | 3.56 (1.38, 9.21) | 0.009 |
| Change effort score | *per 1 point* | 0.49 (0.14, 1.67) | 0.254 |
| Change efficacy score | *per 1 point* | 1.25 (0.50, 3.16) | 0.634 |

^a^ Logistic regression model

^b^ SES = Socioeconomic Status (Index of Relative Socioeconomic Advantage and Disadvantage)

**Additional File 7:** Imbalance across time points in characteristics: odds of providing outcomes at post-program vs pre-program only ^a^

| **Variable** | **Category** | **OR (95% CI)** | ***p*** |  |
| --- | --- | --- | --- | --- |
| ***Staff characteristics*** |  |  |  |  |
| Age | *per 10 years* | 1.03 (0.91, 1.16) | 0.678 |  |
| Work hours per week | *per hour* | 1.01 (0.99, 1.02) | 0.332 |  |
| Body mass index, kg/m2 | 25 to <30 vs <25 kg/m2 | 1.16 (0.83, 1.61) | 0.609 |  |
|  | ≥30 vs <25 kg/m2 | 1.17 (0.80, 1.70) |  |  |
| Female | Yes vs No | 0.92 (0.69, 1.22) | 0.562 |  |
| Non-english speaking background | Yes vs No | 0.92 (0.64, 1.32) | 0.649 |  |
| Fulltime employment | Yes vs No | 1.39 (0.97, 1.99) | 0.070 |  |
| Job classification | Middle management vs employee | 1.14 (0.71, 1.81) | 0.865 |  |
|  | Upper management vs employee | 1.01 (0.74, 1.38) |  |  |
| Job category skill level | 2 vs 1 | 0.58 (0.37, 0.91) | 0.085 |  |
|  | 3 vs 1 | 0.93 (0.67, 1.30) |  |  |
|  | 4 vs 1 | 0.45 (0.15, 1.37) |  |  |
|  | 5 vs 1 | 1.12 (0.72, 1.74) |  |  |
| Post-school education | TAFE / trade certificate / diploma vs none | 1.30 (0.76, 2.22) | 0.482 |  |
|  | University / other tertiary vs none | 1.37 (0.82, 2.27) |  |  |
| Shiftworker | Yes vs no | 1.25 (0.43, 3.59) | 0.682 |  |
| ***Champion characteristics*** |  |  |  |  |
| Champion Age | *per 10 years* | 1.03 (0.82, 1.31) | 0.781 |  |
| Female Champion | Yes vs No | 0.89 (0.48, 1.64) | 0.712 |  |
| Occupational Health & Safety role | Yes vs No | 1.05 (0.60, 1.83) | 0.869 |  |
| Workplace health promotion training | Yes vs No | 1.32 (0.77, 2.26) | 0.317 |  |
| Workplace health promotion experience | Yes vs No | 1.38 (0.81, 2.36) | 0.242 |  |
| Champion Job classification | Middle management vs Employee | 1.29 (0.74, 2.26) | 0.260 |  |
|  | Upper management vs Employee | 1.82 (0.88, 3.78) |  |  |
| Champion State | VIC vs NSW/ACT | 0.72 (0.20, 2.54) | 0.998 |  |
|  | QLD vs NSW/ACT | 0.90 (0.36, 2.24) |  |  |
|  | SA vs NSW/ACT | 0.91 (0.43, 1.90) |  |  |
|  | WA/TAS/NT vs NSW | 0.97 (0.29, 3.19) |  |  |
| Postcode SES (State Percentile) ^b^ | *per 10 percentiles* | 1.11 (0.99, 1.23) | 0.063 |  |
| Postcode SES (Australian Percentile) ^b^ | *per 10 percentiles* | 1.09 (0.98, 1.22) | 0.108 |  |
| ***Team Characteristics*** |  |  |  |  |
| Health Interest (0-4) | *per 1 point* | 1.37 (0.97, 1.93) | 0.072 |  |
| Motivation to sit less (0-4) | *per 1 point* | 1.07 (0.73, 1.57) | 0.735 |  |
| Stress level (0-4) | *per 1 point* | 0.98 (0.60, 1.61) | 0.949 |  |
| Includes regional staff | Yes vs No | 0.70 (0.39, 1.26) | 0.230 |  |
| Includes call centre staff | Yes vs No | 0.26 (0.06, 1.19) | 0.083 |  |
| Currently receiving WPHP program | Yes vs No | 0.81 (0.44, 1.51) | 0.510 |  |
| Main team location (state) | ACT vs NSW | 1.07 (0.45, 2.56) | 0.999 |  |
|  | VIC vs NSW | 0.95 (0.49, 1.87) |  |  |
|  | QLD vs NSW | 1.05 (0.33, 3.35) |  |  |
|  | SA vs NSW | 0.96 (0.34, 2.71) |  |  |
|  | WA/TAS/NT vs NSW | 1.07 (0.45, 2.56) | 0.999 |  |
| ***Organisational/workplace characteristics*** | |  |  |  |
| Organisational readiness |  |  |  |  |
| Context score (0-5) | *per 1 point* | **1.60 (1.05, 2.43)** | **0.030** |  |
| Change effort score (0-1) | *per 1 point* | 0.53 (0.27, 1.02) | 0.058 |  |
| Change efficacy (0-5) | *per 1 point* | 1.14 (0.66, 1.98) | 0.639 |  |
| Blue-collar workplace | Yes vs No | 0.96 (0.54, 1.72) | 0.898 |  |
| Small-medium enterprise | Yes vs No | 1.73 (0.97, 3.08) | 0.064 |  |
| Sector | Non-profit vs public | 2.07 (1.04, 4.15) | 0.119 |  |
|  | Private vs public | 1.21 (0.66, 2.21) |  |  |
| COVID-19 Impact Level | | General vs None | 0.60 (0.20, 1.77) | 0.166 |
|  | | High vs None | 0.38 (0.12, 1.20) |  |

^a^ Mixed effects logistic regression model, with random intercept for workplace

^b^ SES = Socioeconomic Status (Index of Relative Socioeconomic Advantage and Disadvantage)

**Additional File 8**: Comparison of random intercept with random slopes models by Aikake’s Information Criteria (AIC) Bayes information Criteria (BIC) and likelihood ratio chi-square test

| Outcome | n | Random intercept | | | Random slopes | | | Comparison | |
| --- | --- | --- | --- | --- | --- | --- | --- | --- | --- |
|  |  | df | AIC | BIC | df | AIC | BIC | Chi-square (1 df) | p |
| Activity as % of workday |  |  |  |  |  |  |  |  |  |
| Sitting | 2477 | 5 | 6119.00 | 6148.07 | 6 | 6111.51 | 6146.40 | 9.491 | 0.002 |
| Standing | 2477 | 5 | 11583.67 | 11612.74 | 6 | 11570.10 | 11604.99 | 15.57 | 0.000 |
| Moving | 2477 | 5 | 11398.73 | 11427.80 | 6 | 11346.05 | 11380.94 | 54.68 | 0.000 |
| % of sitting in prolonged bouts | 2476 | 5 | 22830.42 | 22859.49 | 6 | 22829.91 | 22864.80 | 2.51 | 0.113 |
| Activity preference alignment | |  |  |  |  |  |  |  |  |
| Sitting | 2422 | 5 | 28114.47 | 28143.43 | 6 | 28116.47 | 28151.23 | 0.00 | >0.999 |
| Standing | 2422 | 5 | 26691.64 | 26720.60 | 6 | 26693.64 | 26728.39 | 0.00 | >0.999 |
| Moving | 2422 | 5 | 26479.70 | 26508.66 | 6 | 26481.70 | 26516.45 | 0.00 | >0.999 |
| Perceptions of culture (0-4) |  |  |  |  |  |  |  |  |  |
| Overall score | 2423 | 5 | 5435.01 | 5463.97 | 6 | 5436.72 | 5471.48 | 0.29 | 0.590 |
| Control over sitting | 2423 | 5 | 3122.33 | 3151.29 | 6 | 3123.07 | 3157.83 | 1.26 | 0.262 |
| Organisation supports choices | 2423 | 5 | 2659.56 | 2688.53 | 6 | 2660.64 | 2695.39 | 0.93 | 0.336 |
| Organisation supports moving | 2423 | 5 | 5939.35 | 5968.32 | 6 | 5939.81 | 5974.57 | 1.54 | 0.214 |
| Role modelling | 2423 | 5 | 6900.77 | 6929.73 | 6 | 6902.77 | 6937.52 | 0.00 | >0.999 |
| Supportive culture | 2423 | 5 | 6711.94 | 6740.91 | 6 | 6713.94 | 6748.70 | 0.00 | >0.999 |
| Part of a team (0-4) | 2367 | 5 | 6636.65 | 6665.50 | 6 | 6638.65 | 6673.27 | 0.00 | >0.999 |
| Engagement (0-4) | 2367 | 5 | 6152.29 | 6181.13 | 6 | 6154.28 | 6188.90 | 0.00 | 0.976 |
|  | 2367 | 5 | 2570.23 | 2599.08 | 6 | 2572.16 | 2606.77 | 0.07 | 0.787 |
| Self-rated job performance (0-7) | 2367 | 5 | 2796.56 | 2825.40 | 6 | 2798.56 | 2833.17 | 0.00 | >0.999 |
| Job satisfaction (0-7) | 2367 | 5 | 12316.37 | 12345.22 | 6 | 12318.15 | 12352.77 | 0.22 | 0.641 |
| Sick Days past 28 days, n | 2367 | 5 | 7214.92 | 7243.76 | 6 | 7216.92 | 7251.53 | 0.00 | >0.999 |
| Stress (0-4) | 2367 | 5 | 4949.40 | 4978.24 | 6 | 4951.40 | 4986.01 | 0.00 | >0.999 |
| Energy score (0-4) | 2367 | 5 | 6492.35 | 6521.20 | 6 | 6493.88 | 6528.49 | 0.47 | 0.491 |
| Creativity (0-4) |  |  |  |  |  |  |  |  |  |
| Self-rated health (0-4) |  |  |  |  |  |  |  |  |  |
| Psychological | 2367 | 5 | 6458.41 | 6487.26 | 6 | 6460.41 | 6495.03 | 0.00 | >0.999 |
| Physical | 2367 | 5 | 6426.72 | 6455.57 | 6 | 6428.72 | 6463.34 | 0.00 | >0.999 |
| Musculoskeletal discomfort (0-10) | |  |  |  |  |  |  |  |  |
| Upper body | 2367 | 5 | 11788.30 | 11817.14 | 6 | 11790.04 | 11824.66 | 0.26 | 0.613 |
| Lower back | 2367 | 5 | 11892.03 | 11920.87 | 6 | 11893.59 | 11928.21 | 0.43 | 0.510 |
| Lower body | 2367 | 5 | 11855.21 | 11884.05 | 6 | 11857.21 | 11891.82 | 0.00 | >0.999 |

Linear mixed models, with random intercepts for workplace and staff (nested in workplace) and default covariance (independent)

**Additional File 9:** Sensitivity analysis excluding pre-program surveys collected after the BeUpstanding program had commenced (k=82 workplaces n=1990-1991 staff) ^a^

| Outcome | Adjusted, excluding late surveys ^b^ | | |
| --- | --- | --- | --- |
|  | n | Change (95% CI) | p |
| Activity as % of workday |  |  |  |
| Sitting ^c,d^ | 1991 | -8.0 (-9.6, -6.4) | <0.001 |
| Standing ^c,e^ | 1991 | 5.7 (3.7, 7.7) | <0.001 |
| Moving ^c,e^ | 1991 | 1.2 (0.0, 2.4) | 0.041 |
| % of sitting in prolonged bouts ^c^ | 1990 | -9.1 (-11.1, -7.1) | <0.001 |
| Activity preference alignment |  |  |  |
| Sitting | 1991 | -34.2 (-40.7, -27.6) | <0.001 |
| Standing | 1991 | -17.9 (-22.7, -13.0) | <0.001 |
| Moving | 1991 | -10.1 (-14.9, -5.3) | <0.001 |
| Perceptions of culture (0-4) |  |  |  |
| Overall culture score | 1991 | 0.37 (0.31, 0.43) | <0.001 |
| Perceived control over sitting ^d^ | 1991 | 0.23 (0.15, 0.30) | <0.001 |
| Organisation supports choices ^d^ | 1991 | 0.37 (0.30, 0.44) | <0.001 |
| Organisation supports moving | 1991 | 0.27 (0.20, 0.34) | <0.001 |
| Role modelling | 1991 | 0.47 (0.38, 0.56) | <0.001 |
| Supportive culture | 1991 | 0.47 (0.39, 0.55) | <0.001 |
| Feeling part of a team (0-4) | 1991 | 0.00 (-0.08, 0.09) | 0.932 |
| Feeling engaged (0-4) | 1991 | 0.03 (-0.04, 0.10) | 0.448 |
| *Productivity and Health* |  |  |  |
| Self-rated job performance (0-7) ^d^ | 1991 | 0.09 (0.01, 0.16) | 0.021 |
| Job satisfaction (0-7) ^d^ | 1991 | 0.07 (-0.01, 0.16) | 0.090 |
| Sick Days past 28 days (0-28) ^e^ | 1991 | 0.00 (0.00, 0.00) | 0.820 |
| Stress (0-4) | 1991 | 0.04 (-0.05, 0.13) | 0.412 |
| Energy score (0-4) | 1991 | 0.12 (0.06, 0.17) | <0.001 |
| Creativity (0-4) | 1991 | 0.00 (-0.08, 0.08) | 0.984 |
| Self-rated health (0-100) |  |  |  |
| Psychological | 1991 | 0.06 (-0.01, 0.13) | 0.102 |
| Physical | 1991 | 0.11 (0.05, 0.18) | <0.001 |
| Musculoskeletal discomfort (0-100) |  |  |  |
| Upper body | 1991 | -0.53 (-0.76, -0.30) | <0.001 |
| Lower back | 1991 | -0.57 (-0.81, -0.33) | <0.001 |
| Lower body | 1991 | -0.34 (-0.58, -0.10) | 0.006 |

^a^ Estimated from linear mixed models, with random intercepts for workplace, and staff (nested within workplace). Table reports contrasts of marginal means, with expressions used for transformed outcomes.

^b^ Adjusts for variables that may be imbalanced across the pre- and post- program respondents: small-medium enterprise (yes/no); inclusion of regional staff (yes/no); inclusion of call-centre staff (yes/no) ; Champion Occupational Health & Safety role (yes/no); COVID-19 impact level (none / general / high) ; public sector (yes/no) ; organisational readiness (context score) ; Champion age (years); full-time employment (yes/no); Job category skill level (1-5); team health interest level (0-4); Postcode SES (State Percentile of Index of Relative Socioeconomic Advantage & Disadvantage)

^c^ Model also includes random slopes for workplace

^d^ Inverse log transformed as ln(1 + maximum value – variable)

^e^ log transformed as ln(variable) for positive or ln (variable + 0.001) for non-negative variables

**Additional File 10**: Satisfaction with the program within priority sectors

|  | Public Sector | Blue-collar workplace | Regional / remote ^a^ | Small business (small-medium enterprise) | Call centre ^a^ |  |
| --- | --- | --- | --- | --- | --- | --- |
| **Champion satisfaction** | n=35 | n=23 | n=21 | n=22 | n=3 |  |
| Easy to run |  |  |  |  |  |  |
| Yes | 25 (71.4%) | 15 (65.2%) | 16 (76.2%) | 20 (90.9%) | 3 (100.0%) |  |
| Somewhat | 9 (25.7%) | 8 (34.8%) | 4 (19.0%) | 2 (9.1%) | 0 (0%) |  |
| No | 1 (2.9%) | 0 (0%) | 1 (4.8%) | 0 (0%) | 0 (0%) |  |
| Met expectations |  |  |  |  |  |  |
| Yes | 27 (77.1%) | 17 (73.9%) | 17 (81.0%) | 18 (81.8%) | 3 (100.0%) |  |
| Somewhat | | 7 (20.0%) | 6 (26.1%) | 4 (19.0%) | 3 (13.6%) | 0 (0%) |
| No | 1 (2.9%) | 0 (0%) | 0 (0%) | 1 (4.5%) | 0 (0%) |  |
| Would recommend program |  |  |  |  |  |  |
| Yes | 29 (82.9%) | 20 (87.0%) | 17 (81.0%) | 20 (90.9%) | 3 (100.0%) |  |
| Maybe | 6 (17.1%) | 3 (13.0%) | 4 (19.0%) | 1 (4.5%) | 0 (0%) |  |
| Probably not | 0 (0%) | 0 (0%) | 0 (0%) | 1 (4.5%) | 0 (0%) |  |
| Definitely not | 0 (0%) | 0 (0%) | 0 (0%) | 0 (0%) | 0 (0%) |  |
| Would run again |  |  |  |  |  |  |
| Probably or definitely yes | 26 (74.3%) | 14 (60.9%) | 13 (61.9%) | 17 (77.3%) | 3 (100.0%) |  |
| Unsure / Maybe | 7 (20.0%) | 6 (26.1%) | 5 (23.8%) | 3 (13.6%) | 0 (0%) |  |
| Probably or definitely not | 2 (5.7%) | 3 (13.0%) | 3 (14.3%) | 2 (9.1%) | 0 (0%) |  |
| Would rollout the program |  |  |  |  |  |  |
| N/A - team was whole small/medium org | 4 (11.4%) | 9 (39.1%) | 5 (23.8%) | 14 (63.6%) | 3 (100.0%) |  |
| Yes or already done | 13 (37.1%) | 7 (30.4%) | 9 (42.9%) | 5 (22.7%) | 0 (0%) |  |
| No | 8 (22.9%) | 0 (0%) | 3 (14.3%) | 0 (0%) | 0 (0%) |  |
| Unsure | 10 (28.6%) | 7 (30.4%) | 4 (19.0%) | 3 (13.6%) | 0 (0%) |  |
| **Staff satisfaction ^b^** | n=288 | n=222 | n=184 | n=207 | n=11 |  |
| Enjoyed BeUpstanding |  |  |  |  |  |  |
| N/A or What’s BeUpstanding? | 13 | 12 | 12 | 6 | 2 |  |
| Yes | 206 (74.9%) | 153 (72.9%) | 135 (78.9%) | 150 (72.5%) | 9 (100.0%) |  |
| No | 17 (6.2%) | 10 (4.8%) | 7 (4.1%) | 10 (4.8%) | 0 (0%) |  |
| Unsure | 52 (18.9%) | 47 (22.4%) | 30 (17.5%) | 41 (19.8%) | 0 (0%) |  |
| Would do program again |  |  |  |  |  |  |
| N/A or What’s BeUpstanding? | 4 | 8 | 6 | 4 | 2 |  |
| Yes | 224 (78.9%) | 151 (70.6%) | 134 (75.3%) | 148 (72.9%) | 9 (100.0%) |  |
| No | 20 (7.0%) | 19 (8.9%) | 13 (7.3%) | 21 (10.3%) | 0 (0%) |  |
| Unsure | 40 (14.1%) | 44 (20.6%) | 31 (17.4%) | 34 (16.7%) | 0 (0%) |  |

^a^ Team included staff from these sectors

^a^ Percentages exclude participants who responded not applicable (N/A) or “What’s BeUpstanding?”)
